# Patterns and Consequences of Care Fragmentation in Post-Surgical Management of Upper Gastrointestinal and Hepatopancreatobiliary Cancers

**DOI:** 10.1245/s10434-025-18052-8

**Published:** 2025-08-20

**Authors:** Melissa Justo, Konmal Ali, Sara Sakowitz, Ayesha Ng, Sona Mahrokhi, Syed Shaheer Ali, Peyman Benharash, Mark D. Girgis

**Affiliations:** Department of Surgery, David Geffen School of Medicine at UCLA, Los Angeles, CA USA

**Keywords:** Care fragmentation, Upper gastrointestinal, Hepatopancreatobiliary, Esophageal cancer, Gastric cancer, Nationwide readmission database

## Abstract

**Background:**

Upper gastrointestinal (UGI) and hepatopancreatobiliary (HPB) oncologic operations are frequently performed at major referral centers. Postoperatively, many patients face care fragmentation (CF), which has been previously linked to inferior outcomes. This analysis examines clinical and financial outcomes of CF following UGI and HPB cancer operations.

**Patients and Methods:**

The 2016–2022 Nationwide Readmissions Database identified adults (≥ 18 years) who underwent UGI and HPB oncologic surgery. Patients readmitted to a nonindex facility within 30 days of discharge comprised the CF cohort. Multivariable models assessed the association of CF with clinical outcomes and identified related factors.

**Results:**

Among 8384 UGI and 16,235 HPB surgical oncology patients, CF affected 15.2% and 13.3%, respectively. CF was associated with higher rates of major adverse events in both groups. Patients undergoing the UGI procedure showed increased odds of respiratory complications (adjusted odds ratio [AOR] 1.67, 95% confidence interval [CI] 1.34, 2.09), while patients undergoing the HPB procedure experienced higher risks of in-hospital mortality (AOR 1.84, 95% CI 1.15–2.94), cardiac (AOR 1.74 95% CI 1.12, 2.71), and respiratory (AOR 2.45, 95% CI 1.87, 3.21) complications. CF was not associated with increased hospitalization costs or longer stays in either cohort.

**Conclusions:**

CF significantly affects postoperative outcomes following UGI and HPB cancer surgeries, with differential impacts between cohorts. The lack of association with increased costs or longer hospital stays may reflect suboptimal care continuity rather than equivalent efficiency. Given CF’s persistent prevalence and clinical significance, these findings highlight the need for enhanced interhospital coordination to improve outcomes for complex oncologic surgical patients.

**Supplementary Information:**

The online version contains supplementary material available at 10.1245/s10434-025-18052-8.

Upper gastrointestinal (UGI) and hepatopancreatobiliary (HPB) cancers represent a diverse group of malignancies that rank among the most common causes of cancer-related deaths worldwide.^1^ Although complete surgical resection remains the gold standard for curative treatment, these operations are technically demanding and carry a substantial risk of complications and rehospitalization.^2^ Given the treatment complexity and associated risks, continuity of care throughout all treatment phases is anticipated to improve outcomes in the recovery phase.

With a large body of evidence supporting a positive operative volume–outcome relationship for complex procedures,^3–5^ growing quality initiatives such as the LEAPFROG Group^6^ have advocated for centralization of high-risk cancer operations to centers meeting specific minimum volume thresholds.^7^ With many patients traveling farther to receive care at “expert centers,” an unintended consequence of centralization has been rehospitalization at a different facility, a phenomenon referred to as “care fragmentation.” In 2016, Zheng et al. linked postoperative CF to adverse outcomes in major cancer operations. Notably, patients receiving liver and pancreatic cancer resections had a nearly threefold increase in the odds of nonindex readmission compared with those with rectal cancer, suggesting the putative influence of procedural complexity on the risk of CF.^8^ Furthermore, previous analyses of postoperative CF in complex oncologic surgery are limited to small, dated cohorts, warranting further investigation at the national level.

The present study examined the association of CF with postoperative clinical and financial outcomes of UGI and HPB oncologic operations. We hypothesized care fragmentation to be associated with increased risk-adjusted odds of mortality and complications during readmission. We further hypothesized that this association would be more pronounced in patients with HPB cancers compared with UGI.

## Patients and Methods

### Data Source and Study Population

**Fig. 1:**
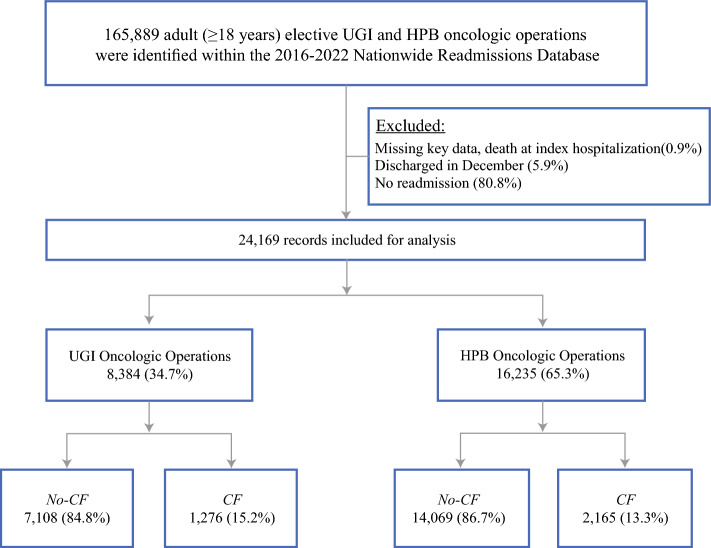
CONSORT flowchart of survey-weighted estimates; a total of 24,169 hospitalization records for elective UGI and HPB oncologic surgery with at least one readmission were identified in the 2016–2022 Nationwide Readmissions Database; *UGI* upper gastrointestinal, *HPB* hepatopancreatobiliary, *CF* care fragmentation, *no-CF* no care fragmentation

All elective adult (≥ 18 years) hospitalizations entailing UGI (gastric and esophageal) and HPB (hepatic, pancreatic, and biliary ductal) oncologic operations were tabulated from the 2016–2022 Nationwide Readmissions Database (NRD). As the largest all-payer readmission database in the USA, the NRD provides patient- and hospital-level data for nearly 60% of all hospitalizations.^9^ Selection criteria for the study cohort are detailed in Fig. 1. Surgical interventions for primary UGI and HPB malignancies included subsets of esophageal, gastric, hepatic, pancreatic, and bile duct cancers, all of which were ascertained via previously validated International Classification of Diseases 10th revision (ICD-10) diagnosis and procedure codes^10^ (Supplementary Table 1). Records missing key data (0.9%) and those without a postoperative readmission (80.8%) were not included for analysis. To ensure sufficient follow-up, discharges in December of each year (5.9%) were also excluded.

### Variable Definitions and Study Outcomes

The NRD Data Dictionary was used to define patient, procedure, and hospital characteristics.^11^ UGI operations included esophageal and gastric cancer resections, while the HPB operations consisted of hepatic, pancreatic, and bile duct cancer resections. Records entailing readmission at a nonindex facility within 30 days of operative discharge comprised the CF cohort (others: No-CF). Relevant patient comorbidities, such as cardiac arrhythmias, diabetes, and hypertension, were identified using previously reported ICD-10 codes.^12^ Postoperative complications were classified as blood-transfusion-related, cardiac, infectious, renal, respiratory, or thromboembolic, as described in prior work.^12^ The van Walraven modification of the Elixhauser Comorbidity Index was employed to quantify the burden of chronic illness.^13^ Facilities were categorized as low-, medium-, and high-volume hospitals on the basis of tertiles of annual UGI and HPB caseloads, respectively. For UGI cancer, the 33rd percentile cutoff was consistently < 3 cases/ year, while the 67th percentile ranged from > 9 to > 12 cases/year. For HPB cancer, the 33rd percentile ranged from < 4 to < 6 cases/year, and the 67th percentile ranged from > 21 to > 28 cases/ year. CF was then classified by index and readmission facility volumes, with “high to low” indicating the index operation occurring at a high-volume hospital and being readmitted a low-volume hospital, and so on. Hospitalization costs were calculated by applying center-specific cost-to-charge ratios to total charges, with inflation adjustment using the 2022 Personal Healthcare Price Index.^14^ Nonhome discharge was defined as transfer to a short-term or skilled nursing facility. The 30-day readmission was defined as a repeat readmission within 30 days of the first rehospitalization.

The primary outcomes of interest were in-hospital mortality and the development of postoperative complications. We secondarily assessed resource utilization including readmission costs, duration of stay (LOS), nonhome discharge, and odds of repeat readmission within 30-days of the first rehospitalization.

### Statistical Analysis

All statistical analyses were performed using Stata 16.1 (StataCorp, College Station, Tx). Statistical significance was set at *α* = 0.05. Categorical variables are presented as percentages (%) while continuous variables are expressed as medians with interquartile range (IQR) or means and standard deviation (SD), as appropriate. Bivariate comparisons were conducted using Pearson’s chi-squared and Mann-Whitney *U* tests, as appropriate. Cuzick’s nonparametric test (nptrend) was employed to assess the significance of temporal trends.^15^ To account for inherent differences between groups, entropy balancing was used to achieve covariate balance,^16^ as previously described. Multivariable models adjusting for relevant patient and hospital characteristics were then developed to determine the independent association of care fragmentation with the outcomes of interest. Receiver-operating characteristics (C-statistic) were computed to evaluate model performance, where applicable. Regression estimates are presented as adjusted odds ratios (AOR) for logistic or beta-coefficients (*β*) for linear models, both with 95% confidence intervals (CI). This study was deemed exempt from full review by the Institutional Review Board at the University of California, Los Angeles.

## Results

### Characteristics of UGI and HPB Surgical Oncologic Patients

**Fig. 2:**
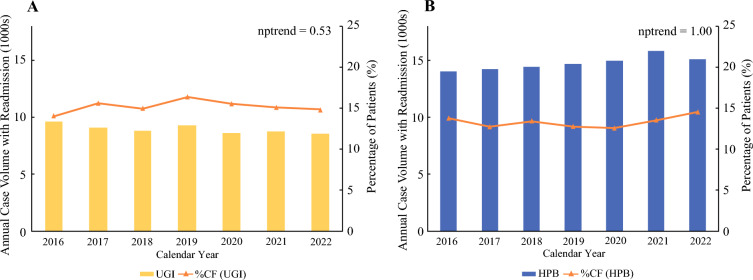
Trends in UGI and HPB oncologic surgery volume and incidence of care fragmentation; case volumes for UGI (**A**) and HPB (**B**) cancer resection, along with the percentage of annual care fragmentation remained stable across the study period (**A**, nptrend = 0.53; **B**, nptrend = 1.00); *CF* care fragmentation, *UGI* upper gastrointestinal, *HPB* hepatopancreatobiliary

Of the 8384 and 16,235 patients undergoing UGI and HPB surgical oncologic procedures, respectively, who met the study criteria, 1276 (15.2%) in the UGI group and 2165 (13.3%) in the HPB group experienced CF (Fig. 1). Over the study period, the proportion of patients experiencing CF in both the UGI and HPB cohorts exhibited minimal change throughout the study period (nptrend = 0.53 and 1.00, respectively, Fig. 2).

In the UGI cohort, patients experiencing CF were younger (66 [59–74] vs. 68 [61–75] years, *P* < 0.001) and more frequently insured by Medicare (61.7 vs. 53.9%, *P* = 0.01) than no-CF patients. In addition, the predominant pattern of CF in the UGI cohort involved index hospitalization at a high-volume center with subsequent readmission to either another high-volume (35.6%) or low-volume (21.9%) center.

Similarly, patients experiencing CF in the HPB cohort more frequently had Medicare coverage (69.2 vs. 56.7%, *P* < 0.001) and were more often readmitted at low- (17.7 vs. 0.9%, *P* < 0.001) and medium- (37.0 vs. 5.2%, *P* < 0.001) volume centers than no-CF patients. However, in contrast to the UGI cohort, patients experiencing CF in the HPB cohort were older (70 [63–76] vs. 67 [60–74] years, *P* < 0.001) and had higher comorbidity burden (4.3 ± 1.7 vs. 3.9 ± 1.7, *P* < 0.001) than no-CF patients. Specifically, congestive heart failure (9.5 vs. 6.5%, *P* < 0.001), diabetes (38.7 vs. 32.7%, *P* < 0.001), and peripheral vascular disease (7.3 vs. 4.3%, *P* < 0.001) were more prevalent in the HPB CF group. All patient, operative, and hospital characteristics for the UGI and HPB cohorts are further presented in Table 1.

**Table 1:** Patient and hospital characteristics

		UGI			HPB	
	No-CF (*n* = 7108)	CF (*n* = 1276)	*P-*value	No-CF (*n* =14,069)	CF (*n* =2165)	*P-*value
Age (years [IQR])	68 [61–75]	66 [59–74]	< 0.001	67 [60–74]	70 [63–76]	< 0.001
Female	27.3	27.0	0.86	44.0	43.9	0.92
Elixhauser comorbidity index (mean ± SD)	4.0 ± 1.7	4.2 ± 1.6	0.04	3.9 ± 1.7	4.3 ±1.7	< 0.001
*Community income percentile*			0.27			0.14
> 75th	25.0	22.4		27.7	24.5	
51–75th	25.2	24.4		27.6	26.6	
26–50th	27.0	30.7		24.3	26.3	
0–25th	22.8	22.5		20.5	22.6	
*Insurance coverage*			0.01			< 0.001
Private	33.4	28.0		32.9	23.0	
Medicare	53.9	61.7		56.7	69.2	
Medicaid	8.2	7.6		6.6	5.1	
Other payer	4.4	2.7		3.7	2.7	
*Comorbidities*						
Arrhythmia	31.3	34.4	0.12	22.5	26.1	0.02
Chronic pulmonary disease	18.0	21.3	0.06	14.7	17.8	0.01
Coagulopathy	8.1	8.0	0.92	8.4	8.1	0.67
Congestive heart failure	8.7	9.3	0.67	6.5	9.5	< 0.001
Diabetes	26.0	24.4	0.42	32.7	38.7	< 0.001
Hypertension	55.6	50.9	0.03	57.3	61.7	0.02
Late-stage chronic kidney disease	1.1	0.9	0.72	0.9	1.9	0.01
Liver disease	5.8	6.5	0.50	13.0	15.7	0.03
Peripheral vascular disease	5.9	5.7	0.83	4.3	7.3	< 0.001
Obesity	14.0	15.1	0.50	15.0	15.9	0.5
*Surgical approach*			0.70			0.3
Open	59.4	58.5		84.2	82.9	
Minimally invasive	40.6	41.5		15.8	17.1	
*Index case*			0.34			
Esophageal CA						
Esophageal excision	17.3	15.7		–	–	–
Esophagectomy	11.5	13.4		–	–	–
Colon interposition	0.3	0.1		–	–	–
Jejunal graft	0.1	0.0		–	–	–
Unspecified autologous replacement	0.3	0.0		–	–	–
*Gastric CA*						
Excision of esophagogastric junction	9.6	10.1		–	–	–
Esophagogastrectomy	6.2	6.7		–	–	–
Total gastrectomy	37.8	41.0		–	–	–
Partial gastrectomy	14.5	10.9		–	–	–
Antrectomy	0.6	0.6		–	–	–
Pylorogastrectomy/distal gastrectomy	1.8	1.5		–	–	–
*Hepatic CA*						0.001
Hepatic wedge resection	–	–	–	10.6	19.1	
Right hepatic lobectomy	–	–	–	3.0	4.1	
Left hepatic lobectomy	–	–	–	1.8	1.7	
*Pancreatic CA*						
Partial pancreatectomy	–	–	–	64.5	55.6	
Total pancreatectomy	–	–	–	2.6	3.6	
*Biliary ductal CA*						
Excision/resection of common bile duct	–	–	–	16.4	15.0	
Excision/resection of cystic duct	–	–	–	0.2	0.1	
Excision/resection of ampulla of vater	–	–	–	0.2	0.3	
Excision/destruction right hepatic duct	–	–	–	0.3	0.2	
Excision/destruction left hepatic duct	–	–	–	0.3	0.1	
*Hospital teaching status for readmission*			0.57			0.3
Non-metropolitan	1.0	1.1		0.7	0.9	
Metropolitan nonteaching	6.6	5.5		4.3	5.2	
Metropolitan teaching	92.4	93.4		95.1	93.9	
*Hospital volume for index hospitalization*			< 0.001			0.43
Low	3.8	2.0		0.7	1.2	
Medium	13.2	9.2		6.5	6.4	
High	83.0	88.8		92.7	92.4	
*Hospital volume for readmission*			< 0.001			< 0.001
Low	3.2	20.8		0.9	17.7	
Medium	10.2	35.1		5.2	37.0	
High	86.6	44.1		94.0	45.3	
*CF classification*			< 0.001			< 0.001
Low-to-low	1.2	0.9		1.2	0.8	
Low-to-high	0.2	0.8		0.5	1.2	
High-to-low	1.7	21.9		0.2	16.8	
High-to-high	78.8	35.6		81.1	34.3	

Reported as percentages, unless otherwise noted. Statistical significance was set at *α* < 0.05.

*CF* care fragmentation, *no-CF* no care fragmentation, *IQR* interquartile range, *SD* standard deviation

Our results exhibit a dominance of “high-to-high” (35.6 and 34.3%, Table 1) and “high-to-low” (21.9 and 16.8%, Table 1) patterns of CF among fragmented patients in the UGI and HPB cohorts, respectively. However, there are minimal “low-to-high” volume transitions (0.8 and 1.2%)

### Unadjusted Clinical Outcomes

Upon readmission, patients experiencing CF among the UGI cohort were not found to have a significant difference in in-hospital mortality (5.2 vs. 4.5%, *P* = 0.46, Table 2). Compared with no-CF patients, those experiencing CF in the UGI cohort were more commonly readmitted for respiratory complications (27.4 vs. 16.8%, *P* < 0.001) but incurred lower hospitalization costs ($11.5 k [$6.9–22.2 k] vs. $14.4 k [$7.8–30.6 k], *P* = 0.01), shorter length of stay (4 [2–7] vs. 6 [3–11] days, *P* < 0.001). However, patients experiencing CF were more likely to require nonhome discharge (29.0 vs. 20.9%, *P* = 0.001) than others.

**Table 2:** Risk-adjusted outcomes of readmission

	UGI			HPB		
	Estimate (AOR/*β*)	95% CI	*P*-value	Estimate (AOR/*β*)	95% CI	*P*-value
*Clinical Outcomes*						
In-hospital mortality (%)	1.02	0.64, 1.64	0.91	1.84	1.15, 2.94	0.01
Additional complications (%)						
Blood transfusion	0.92	0.66, 1.29	0.63	1.11	0.86, 1.43	0.43
Cardiac	1.43	0.86, 2.39	0.17	1.74	1.12, 2.71	0.01
Infectious	0.79	0.63, 0.98	0.03	0.96	0.81, 1.15	0.68
Renal	1.10	0.82, 1.46	0.51	1.39	1.13, 1.71	0.002
Respiratory	1.67	1.34, 2.09	< 0.001	2.45	1.87, 3.21	< 0.001
Thromboembolic	1.10	0.72, 1.70	0.66	1.32	0.94, 1.87	0.11
*Resource utilization*						
Cost (USD $1000s, median [IQR])	+ 5.50	− 6.86, + 17.87	0.38	+ 0.31	− 3.78, +4.40	0.88
Length of stay (days, median [IQR])	+ 1.27	− 1.87, +4.41	0.43	−0.79	− 1.77, + 0.19	0.12
30-day readmission (%)	1.18	0.97, 1.44	0.11	0.98	0.82, 1.18	0.86
Nonhome discharge (%)	1.40	1.09, 1.78	0.01	1.63	1.32, 2.01	< 0.001

Logistic model outputs are reported as adjusted odds ratios (AOR) with 95% confidence intervals (95% CI).

*AOR* adjusted odds ratio, *CI* confidence interval, *USD* United States dollar

Patients experiencing CF among the HPB cohort more commonly experienced in-hospital mortality (5.6 vs. 2.5%, *P* < 0.001) than no-CF patients. They also more frequently developed cardiac (5.5 vs. 2.5%, *P* < 0.001), renal (21.2 vs. 14.9%, *P* < 0.001), and respiratory (15.8 vs. 6.2%, *P* < 0.001) complications upon readmission. Similar to those of the UGI cohort, patients experiencing CF in the HPB cohort also had decreased hospitalization costs ($11.6 k [$6.9–21.5 k] vs. $12.8 k [$7.2–24.7 k], *P* < 0.001), shorter length of stay (4 [2–7] vs. 5 days [3–9], *P* < 0.001) and more frequent nonhome discharge (27.8 vs. 16.4%, *P* < 0.001).

### Risk-Adjusted Clinical Outcomes

Following risk adjustment (model C-statistic: 0.63), the odds of mortality were comparable between CF and no-CF patients in the UGI cohort. Apart from CF being significantly associated with increased odds of respiratory complications (AOR 1.67, 95% CI 1.34, 2.09), most complications at readmission were comparable between CF and no-CF (Table 3) groups. In addition, CF was associated with an increased rate of nonhome discharge in the UGI cohort.

**Table 3:** Factors associated with care fragmentation

	UGI			HPB		
	Estimate (AOR)	95% CI	*P*-value	Estimate (AOR)	95% CI	*P*-value
*Demographics*						
Age (per year)	1.01	0.99, 1.02	0.34	1.01	1.00, 1.02	0.01
Female (ref.: male)	0.97	0.77, 1.23	0.81	0.89	0.75, 1.06	0.12
Elixhauser comorbidity index (per unit)	1.07	0.97, 1.18	0.20	1.02	0.95, 1.10	0.56
*Income percentile*						
> 75th	Ref.	–	–	Ref.	–	–
51–75th	1.00	0.75, 1.35	0.97	1.06	0.85, 1.32	0.62
26–50th	1.17	0.87, 1.56	0.30	1.03	0.81, 1.30	0.82
< 25th	0.99	0.72, 1.37	0.98	0.97	0.76, 1.25	0.82
*Insurance coverage*						
Private insurance	Ref.	–	–	Ref.	–	–
Medicare	1.01	0.75, 1.36	0.93	1.29	1.01, 1.44	0.04
Medicaid	0.85	0.56, 1.30	0.46	1.10	0.74, 1.62	0.64
Other payer	0.75	0.40, 1.39	0.36	1.15	0.68, 1.94	0.61
*Comorbidities*						
Arrhythmia	1.11	0.86, 1.42	0.42	1.17	0.94, 1.44	0.16
Chronic pulmonary disease	0.95	0.71, 1.27	0.74	1.15	0.90, 1.45	0.26
Diabetes	0.79	0.60, 1.05	0.11	1.17	0.96, 1.45	0.11
Peripheral vascular disease	0.90	0.56, 1.43	0.65	1.33	0.93, 1.88	0.12
*Surgical approach*						
Open	Ref.	–	–	Ref.	–	–
Minimally invasive	1.15	0.86, 2.07	0.20	1.22	0.97, 1.52	0.09
*Hospital teaching status*						
Non-metropolitan	Ref.	–	–	Ref.	–	–
Metropolitan non-teaching	0.75	0.26, 2.20	0.22	0.49	0.20, 1.21	0.12
Metropolitan teaching	0.89	0.32, 2.46	0.82	0.51	0.21, 1.20	0.12
*Hospital volume for index operation*						
Low	Ref.	–	–	Ref.	–	–
Medium	1.17	0.85, 1.61	0.35	1.04	0.78, 1.39	0.78
High	1.26	0.94, 1.69	0.12	1.21	0.93, 1.57	0.16

Patient- and hospital-level variables identified as factors associated with CF stratified by subsect of surgical oncologic surgery: UGI or HPB. Logistic model outputs are reported as adjusted odds ratios (AOR) with 95% confidence intervals (95% CI).

*CF* care fragmentation, *AOR* adjusted odds ratio, *CI* confidence interval, *UGI* upper gastrointestinal, *HPB* hepatopancreatobiliary

**Fig. 3:**
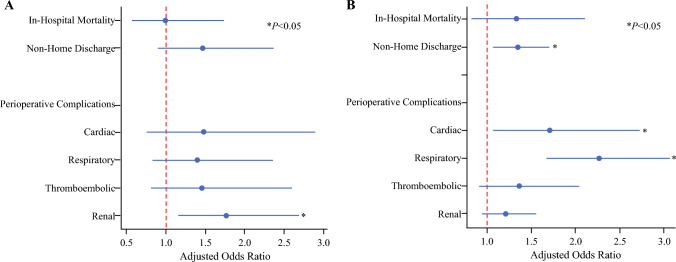
Outcomes of care fragmentation; risk-adjusted readmission outcomes following upper gastrointestinal (**A**) and hepatopancreatobiliary (**B**) oncologic surgery for care fragmentation; *indicates statistical significance, *P* < 0.05; error bars represent 95% confidence intervals

After risk adjustment (model C-statistic: 0.69), CF was significantly associated with greater odds of mortality (AOR 1.84, 95% CI 1.15, 2.94) as well as cardiac (AOR 1.74 95% CI 1.12, 2.71), and respiratory (AOR 2.45, 95% CI 1.87, 3.21) complications in the HPB cohort. Similar to UGI, CF in the HPB cohort was linked to a greater likelihood of nonhome discharge (AOR 1.35, 95% CI 1.07, 1.70) (Fig. 3).

We performed subgroup analyses by UGI and HPB surgery subtype but found no significant differential effects of care fragmentation on outcomes, although sparse data in rare procedures limited interpretability and larger studies are needed to definitively assess procedure-specific associations (Supplementary Table 3). In addition, we analyzed mortality associations stratified by hospital volume transitions and found nonsignificant trends, suggesting CF may have differential effects on the basis of volume changes (Supplementary Table 4), but larger studies are needed to confirm these volume-dependent associations.

### Factors Associated with Care Fragmentation

For the UGI cohort, none of the patient characteristics tested exhibited significant associations with the development of care fragmentation (Table 3). Among patients in the HPB cohort, older age (AOR 1.01/year, 95% CI 1.01–1.02) and Medicare coverage (AOR 1.29, 95% CI 1.01–1.44) were identified as drivers of CF. Patients who had their index operation at a high-volume hospital were much more likely to experience CF upon readmission in both the UGI (AOR 31.28, 95% CI 16.21, 60.34) and HPB cohorts (AOR 15.99, 95% CI 7.74, 33.02).

## Discussion

In this national analysis, care fragmentation affected 15.2 and 13.3% of all 30-day readmissions following UGI and HPB oncologic procedures, respectively. Patients facing fragmented care exhibited a higher prevalence of comorbidities and socioeconomic risk factors that may have predisposed them to adverse clinical and financial outcomes. Despite accounting for these underlying variables, CF remained independently associated with higher in-hospital morbidity in the HPB cohort. Interestingly, CF was not associated with increased hospitalization expenses and longer hospital stays within either cohort. Given the prevalence, clinical significance, and economic burden of CF, these findings warrant further investigation.

This study revealed care fragmentation rates among UGI and HPB surgical oncologic readmissions consistent with previously published findings.^17,18^ Of note, the proportion of patients experiencing CF within each cohort exhibited no significant change across the 6-year study period (Fig. 2). Though it is well established that CF is associated with greater odds of in-hospital mortality and postoperative complications,^8,17^ existing structural relationships between hospitals may be reinforcing fragmentation. Our results exhibit a dominance of “high-to-high” (35.6 and 34.3%, Table 1) and “high-to-low” (21.9 and 16.8%, Table 1) patterns of CF among fragmented patients in the UGI and HPB cohorts, respectively. However, there are minimal “low-to-high” volume transitions (0.8 and 1.2%), suggesting potential barriers in the upward referral pathway from low-volume to high-volume hospitals. These patterns may reflect complex institutional relationships that are driven by reimbursement structures or care coordination challenges.^19^ Policy interventions might need to specifically address these established patterns to effectively reduce fragmentation and its negative outcomes.

**Fig. 4:**
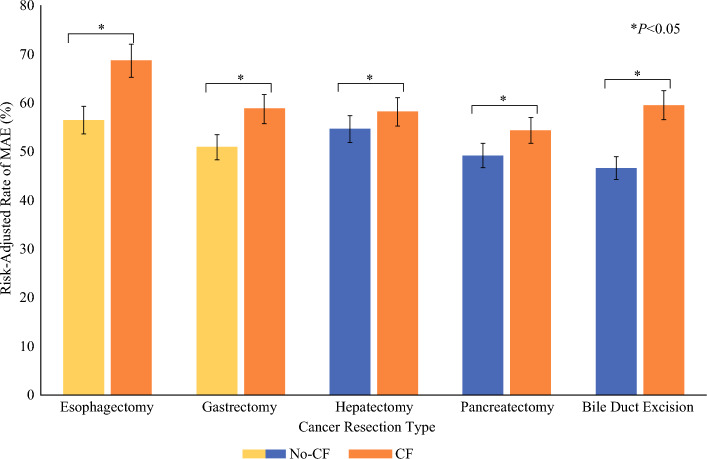
Outcomes of care fragmentation by operative intervention; care fragmentation was consistently associated with higher rates of major adverse events across all UGI and HPB oncologic operations; error bars represent 95% confidence intervals; *CF* care fragmentation, *no-CF* no care fragmentation, *MAE* major adverse events, *UGI* upper gastrointestinal, *HPB* hepatopancreatobiliar

In the present work, CF was consistently associated with higher rates of major adverse events across each type of cancer resection analyzed (Fig. 4). Although the inherent nature of each oncologic procedure predisposes patients to specific complications, studies have demonstrated that CF amplifies their frequency of occurring.^8,17^ Oncologic patients, who frequently present with multiple comorbidities and complex medication regimens, are particularly susceptible to the adverse outcomes of disrupted care communication inherent in CF.^20,21^ In fact, prior investigations have documented associations between CF and increased rates of inappropriate pharmacotherapy^22,23^ as well as suboptimal chronic disease management.^24^ Notably, the detrimental impact of CF manifested more prominently in HPB procedures compared with UGI. This disparity likely stems from the greater procedural complexity, higher comorbidity burden, and increased social vulnerability observed within the HPB patient cohort.

Our findings indicate that CF is not associated with increased hospitalization costs or longer stays in either the UGI or HPB cohorts. While this contrasts with prior studies showing the economic burden of CF,^25,26^ similar inconsistencies have been observed in surgical populations. For instance, nonindex readmissions were linked to shorter LOS and lower costs following HPB surgery and aneurysm repair.^18,27^ This may reflect differences in post-acute care delivery, as patients experiencing CF are often readmitted to lower-volume centers, which may either offer less intensive care or transfer patients to higher-level facilities, thereby reducing documented LOS and costs. However, these metrics may not accurately reflect illness severity. Notably, CF in the HPB cohort was associated with a 1.6-fold increased likelihood of nonhome discharge, a common outcome in oncologic surgery owing to needs for adjuvant therapy, home healthcare, or rehabilitation.^28,29^ CF may compound discharge planning challenges, particularly when coordinated outpatient care is lacking.^30^

Mitigating the adverse effects of CF requires systems-based strategies that enhance interhospital care coordination and ensure continuity of care. Seamless data exchange between institutions can help identify high-risk patients, enable timely interventions, and support targeted discharge and follow-up plans. Digital care platforms that centralize medical records, coordinate appointments, and facilitate interinstitutional communication show promise.^31^ Regional postoperative acute care clinics offer another solution by extending institutional continuity through geographic expansion. For example, Memorial Sloan Kettering Cancer Center successfully implemented regional symptom care clinics to provide safe and accessible postoperative evaluation.^32^ While such models are not new to oncology,^33,34^ they have rarely been applied to acute postoperative care.

The results of this study should be viewed within the scope of several limitations. Given the retrospective design of the study, causal relationships cannot be inferred. As an administrative healthcare database, the NRD lacks granular clinical information required for precise evaluation of each patient’s clinical condition and reasons for readmission, limiting our ability to fully adjust for unmeasured confounders such as cancer staging and performance status. Geographic distance data between patient residence and facilities is also not available, precluding evaluation of whether fragmentation represents necessary versus potentially avoidable care discontinuity. Furthermore, it is commonly acknowledged that ICD-10 diagnosis and procedure coding may vary considerably between hospitals participating in the NRD. Despite these limitations, we believe the present work provides important insights that help fill a gap in existing literature. In addition, we employed rigorous statistical methods and utilized the largest, all-payer readmissions database to strengthen the generalizability of our findings.

In conclusion, our national analysis reveals that CF significantly increases morbidity after UGI and HPB oncologic resections, with patients undergoing HPB procedures experiencing more severe adverse effects likely owing to their higher procedural complexity, comorbidity burden, and social vulnerability. Despite demonstrating no impact on costs or length of stay in either cohort, CF likely reflects substandard care continuity rather than comparable efficiency. Our findings suggest the need for quality improvement initiatives designed to reduce the development and consequences of CF, such as interhospital information exchange systems and longitudinal postoperative care in the outpatient setting.

## Supplementary Information

Below is the link to the electronic supplementary material.

Supplementary file 1 (DOCX 22 KB)

Supplementary file 2 (DOCX 22 KB)

Supplementary file 3 (DOCX 17 KB)

Supplementary file 4 (DOCX 21 KB)

Supplementary file 5 (DOCX 17 KB)

## References

[1] Arnold M, Abnet CC, Neale RE, et al. Global burden of 5 major types of gastrointestinal cancer. *Gastroenterology*. 2020;159(1):335-349.e15. 10.1053/j.gastro.2020.02.068. 32247694 10.1053/j.gastro.2020.02.068 PMC8630546

[2] Schneider EB, Hyder O, Wolfgang CL, et al. Patient readmission and mortality after surgery for hepato-pancreato-biliary malignancies. *J Am Coll Surg*. 2012;215(5):607–15. 10.1016/j.jamcollsurg.2012.07.007. 22921328 10.1016/j.jamcollsurg.2012.07.007 PMC4051393

[3] Luft HS, Bunker JP, Enthoven AC. Should operations be regionalized? The empirical relation between surgical volume and mortality. *N Engl J Med*. 1979;301(25):1364–9. 10.1056/NEJM197912203012503. 503167 10.1056/NEJM197912203012503

[4] Birkmeyer JD, Siewers AE, Finlayson EVA, et al. Hospital volume and surgical mortality in the United States. *N Engl J Med*. 2002;346(15):1128–37. 10.1056/NEJMsa012337. 11948273 10.1056/NEJMsa012337

[5] Reames BN, Ghaferi AA, Birkmeyer JD, Dimick JB. Hospital volume and operative mortality in the modern era. *Ann Surg*. 2014;260(2):244–51. 10.1097/SLA.0000000000000375. 24368634 10.1097/SLA.0000000000000375 PMC4069246

[6] Milstein A, Galvin RS, Delbanco SF, Salber P, Buck CR. Improving the safety of health care: the LEAPFROG initiative. *Eff Clin Pract*. 2000;3(6):313–6. 11151534

[7] Urbach DR. Pledging to eliminate low-volume surgery. *N Engl J Med*. 2015;373(15):1388–90. 10.1056/NEJMp1508472. 26444728 10.1056/NEJMp1508472

[8] Zheng C, Habermann EB, Shara NM, et al. Fragmentation of care after surgical discharge: non-index readmission after major cancer surgery. *J Am Coll Surg*. 2016;222(5):780-789.e2. 10.1016/j.jamcollsurg.2016.01.052. 27016905 10.1016/j.jamcollsurg.2016.01.052 PMC5244824

[9] NRD Overview. Accessed 2 May 2025. https://hcup-us.ahrq.gov/nrdoverview.jsp

[10] Abreu AA, Meier J, Alterio RE, et al. Association of race, demographic and socioeconomic factors with failure to rescue after hepato-pancreato-biliary surgery in the United States. *HPB*. 2024;26(2):212–23. 10.1016/j.hpb.2023.10.001. 37863740 10.1016/j.hpb.2023.10.001

[11] NRD Description of Data Elements. Accessed 2 May 2025. https://hcup-us.ahrq.gov/db/nation/nrd/nrddde.jsp

[12] Madrigal J, Mukdad L, Han AY, et al. Impact of hospital volume on outcomes following head and neck cancer surgery and flap reconstruction. *Laryngoscope*. 2022;132(7):1381–7. 10.1002/lary.29903. 34636433 10.1002/lary.29903

[13] Van Walraven C, Austin PC, Jennings A, Quan H, Forster AJ. A modification of the elixhauser comorbidity measures into a point system for hospital death using administrative data. *Med Care*. 2009;47(6):626–33. 10.1097/MLR.0b013e31819432e5. 19433995 10.1097/MLR.0b013e31819432e5

[14] Cost-to-Charge Ratio Files. Accessed 2 May 2025. https://hcup-us.ahrq.gov/db/ccr/costtocharge.jsp

[15] Cuzick J. A wilcoxon-type test for trend. *Stat Med*. 1985;4(1):87–90. 10.1002/sim.4780040112. 3992076 10.1002/sim.4780040112

[16] Hainmueller J. Entropy balancing for causal effects: a multivariate reweighting method to produce balanced samples in observational studies. *Polit Anal*. 2012;20(1):25–46. 10.1093/pan/mpr025.

[17] Zafar SN, Shah AA, Channa H, Raoof M, Wilson L, Wasif N. Comparison of rates and outcomes of readmission to index vs nonindex hospitals after major cancer surgery. *JAMA Surg*. 2018;153(8):719. 10.1001/jamasurg.2018.0380. 29641833 10.1001/jamasurg.2018.0380 PMC6142950

[18] Beal EW, Bagante F, Paredes A, et al. Index versus non-index readmission after hepato-pancreato-biliary surgery: where do patients go to be readmitted? *J Gastrointest Surg*. 2019;23(4):702–11. 10.1007/s11605-018-3882-y. 30039444 10.1007/s11605-018-3882-y

[19] Dillon EC, Martinez MC, Li M, et al. “It is not the fault of the health care team - It is the way the system works:” a mixed-methods quality improvement study of patients with advanced cancer and family members reveals challenges navigating a fragmented healthcare system and the administrative and financial burdens of care. *BMC Health Serv Res*. 2024;24(1):1378. 10.1186/s12913-024-11744-z. 39529059 10.1186/s12913-024-11744-z PMC11552108

[20] Kern LM, Safford MM, Slavin MJ, et al. Patients’ and providers’ views on causes and consequences of healthcare fragmentation in the ambulatory setting: a qualitative study. *J Gen Intern Med*. 2019;34(6):899–907. 10.1007/s11606-019-04859-1. 30783883 10.1007/s11606-019-04859-1 PMC6544669

[21] Turner JP, Kantilal K, Kantilal K, Holmes HM, Koczwara B. Optimising medications for patients with cancer and multimorbidity: the case for deprescribing. *Clin Oncol (R Coll Radiol)*. 2020;32(9):609–17. 10.1016/j.clon.2020.05.015. 32563549 10.1016/j.clon.2020.05.015

[22] Chu HY, Chen CC, Cheng SH. Continuity of care, potentially inappropriate medication, and health care outcomes among the elderly: evidence from a longitudinal analysis in Taiwan. *Med Care*. 2012;50(11):1002–9. 10.1097/MLR.0b013e31826c870f. 23047791 10.1097/MLR.0b013e31826c870f

[23] Prior A, Vestergaard CH, Vedsted P, et al. Healthcare fragmentation, multimorbidity, potentially inappropriate medication, and mortality: a Danish nationwide cohort study. *BMC Med*. 2023;21(1):305. 10.1186/s12916-023-03021-3. 37580711 10.1186/s12916-023-03021-3 PMC10426166

[24] Maciejewski ML, Hammill BG, Bayliss EA, et al. Prescriber continuity and disease control of older adults. *Med Care*. 2017;55(4):405–10. 10.1097/MLR.0000000000000658. 27755393 10.1097/MLR.0000000000000658 PMC5352484

[25] Van Walraven C, Oake N, Jennings A, Forster AJ. The association between continuity of care and outcomes: a systematic and critical review. *Eval Clin Pract*. 2010;16(5):947–56. 10.1111/j.1365-2753.2009.01235.x. 10.1111/j.1365-2753.2009.01235.x 20553366

[26] Snow K, Galaviz K, Turbow S. Patient outcomes following interhospital care fragmentation: a systematic review. *J Gen Intern Med*. 2020;35(5):1550–8. 10.1007/s11606-019-05366-z. 31625038 10.1007/s11606-019-05366-z PMC7210367

[27] Glebova NO, Hicks CW, Taylor R, et al. Readmissions after complex aneurysm repair are frequent, costly, and primarily at nonindex hospitals. *J Vasc Surg*. 2014;60(6):1429–37. 10.1016/j.jvs.2014.08.092. 25316154 10.1016/j.jvs.2014.08.092

[28] Heid CA, Khoury MK, Thornton MA, Geoffrion TR, De Hoyos AL. Risk factors for non-home discharge following esophagectomy for neoplastic disease. *Ann Thorac Surg*. 2021;111(4):1118–24. 10.1016/j.athoracsur.2020.06.066. 32866477 10.1016/j.athoracsur.2020.06.066 PMC9295198

[29] Avery KNL, Richards HS, Portal A, et al. Developing a real-time electronic symptom monitoring system for patients after discharge following cancer-related surgery. *BMC Cancer*. 2019;19(1):463. 10.1186/s12885-019-5657-6. 31101017 10.1186/s12885-019-5657-6 PMC6524308

[30] Turbow SD, Ali MK, Culler SD, et al. Association of fragmented readmissions and electronic information sharing with discharge destination among older adults. *JAMA Netw Open*. 2023;6(5):e2313592. 10.1001/jamanetworkopen.2023.13592. 37191959 10.1001/jamanetworkopen.2023.13592 PMC10189568

[31] Hopstaken JS, Verweij L, van Laarhoven CJHM, Blijlevens NMA, Stommel MWJ, Hermens RPMG. Effect of digital care platforms on quality of care for oncological patients and barriers and facilitators for their implementation: systematic review. *J Med Internet Res*. 2021;23(9):e28869. 10.2196/28869. 34559057 10.2196/28869 PMC8501408

[32] Brauer DG, Gonen M, Drebin JA, Groeger JS, Jewell EL. Establishing regionalized acute care across a health care system to decentralize postoperative care after oncologic surgery. *JCO Oncol Pract*. 2024;20(5):666–72. 10.1200/OP.23.00392. 38295332 10.1200/OP.23.00392 PMC11657752

[33] Bruera E, Michaud M, Vigano A, Neumann CM, Watanabe S, Hanson J. Multidisciplinary symptom control clinic in a cancer center: a retrospective study. *Support Care Cancer*. 2001;9(3):162–8. 10.1007/s005200000172. 11401100 10.1007/s005200000172

[34] Strasser F, Sweeney C, Willey J, Benisch-Tolley S, Palmer JL, Bruera E. Impact of a half-day multidisciplinary symptom control and palliative care outpatient clinic in a comprehensive cancer center on recommendations, symptom intensity, and patient satisfaction: a retrospective descriptive study. *J Pain Symptom Manage*. 2004;27(6):481–91. 10.1016/j.jpainsymman.2003.10.011. 15165646 10.1016/j.jpainsymman.2003.10.011

