# Supplementary material for: Patterns and Consequences of Care Fragmentation in Post-Surgical Management of Upper Gastrointestinal and Hepatopancreatobiliary Cancers
Source: Ann Surg Oncol. 2025 Aug 20;33(1):568–77. doi: 10.1245/s10434-025-18052-8 (PMC12689737; doi:10.1245/s10434-025-18052-8)
Supplement: Supplementary file 1 — (DOCX 22 KB) [file 10434_2025_18052_MOESM1_ESM.docx]

|  | **International Classification of Disease Codes**  **10^th^ Revision** | |
| --- | --- | --- |
| ***Diagnoses*** |  | |
| Esophageal Cancer |  | C15 D001 Z8501 |
| Gastric Cancer |  | C153 C154 C155 C158 C159 C16 D001 D002 Z8501 Z8502 C7A092 C7A094 |
| Liver Cancer |  | C22 C7A095 |
| Pancreatic Cancer |  | C24 C25 C7A1 C7A8 C170 C7A094 |
| Biliary Ductal Cancer |  | C24 |

| ***Procedures*** |  |
| --- | --- |
| *Esophageal Cancer Resection* |  |
| Excision of Esophagus Open | 0DB50ZZ |
| Excision of Esophagus Percutaneous Endoscopic | 0DB54ZZ |
| Excision of Upper Esophagus Open | 0DB10ZZ |
| Excision of Upper Esophagus Percutaneous Endoscopic | 0DB14ZZ |
| Excision of Middle Esophagus Open | 0DB20ZZ |
| Excision of Middle Esophagus Percutaneous Endoscopic | 0DB24ZZ |
| Excision of Lower Esophagus Open | 0DB30ZZ |
| Excision of Lower Esophagus Percutaneous Endoscopic | 0DB34ZZ |
| Resection of Esophagus Open | 0DT50ZZ |
| Resection of Esophagus Percutaneous Endoscopic | 0DT54ZZ |
| Resection of Upper Esophagus | 0DT10ZZ |
| Resection of Upper Esophagus | 0DT14ZZ |
| Resection of Middle Esophagus Open | 0DT20ZZ |
| Resection of Middle Esophagus Percutaneous Endoscopic | 0DT24ZZ |
| Resection of Lower Esophagus Open | 0DT30ZZ |
| Resection of Lower Esophagus Percutaneous Endoscopic | 0DT34ZZ |
| Replacement of Esophagus with Autologous Tissue Substitute Open | 0DR507Z |
| Replacement of Esophagus with Autologous Tissue Substitute Percutaneous Endoscopic | 0DR547Z |
| Transfer Large Intestine to Esophagus Open | 0DXE0Z5 |
| Transfer Large Intestine to Esophagus Percutaneous Endoscopic | 0DXE4Z5 |
| Transfer Small Intestine to Esophagus Open | 0DX80Z5 |
| Transfer Small Intestine to Esophagus Percutaneous Endoscopic | 0DX84Z5 |
|  |  |
| *Gastric Cancer Resection* |  |
| Excision of Esophagogastric Junction, Open | 0DB40ZZ |
| Excision of Esophagogastric Junction, Percutaneous | 0DB43ZZ |
| Excision of Esophagogastric Junction, Percutaneous  Endoscopic | 0DB44ZZ |
| Excision of Esophagogastric Junction, Natural or Artificial Opening | 0DB47ZZ |
| Excision of Esophagogastric Junction, Natural or Artificial Opening Endoscopic | 0DB48ZZ |
| Resection of Esophagogastric Junction, Open | 0DT40ZZ |
| Resection of Esophagogastric Junction, Percutaneous Endoscopic | 0DT44ZZ |
| Resection of Esophagogastric Junction, Natural or Artificial Opening | 0DT47ZZ |
| Resection of Esophagogastric Junction, Natural or Artificial Opening Endoscopic | 0DT48ZZ |
| Excision of Stomach, Open | 0DB60ZZ |
| Excision of Stomach, Percutaneous | 0DB63ZZ |
| Excision of Stomach, Percutaneous Endoscopic | 0DB64ZZ |
| Excision of Stomach, Natural or Artificial Opening | 0DB67ZZ |
| Excision of Stomach, Natural or Artificial Opening Endoscopic | 0DB68ZZ |
| Resection of Stomach, Open | 0DT60ZZ |
| Resection of Stomach, Percutaneous Endoscopic | 0DT64ZZ |
| Resection of Stomach, Natural or Artificial Opening | 0DT67ZZ |
| Resection of Stomach, Natural or Artificial Opening Endoscopic | 0DT68ZZ |
| Resection of Stomach, Pylorus, Open | 0DT70ZZ |
| Resection of Stomach, Pylorus, Percutaneous Endoscopic | 0DT74ZZ |
| Resection of Stomach, Pylorus, Natural or Artificial Opening | 0DT77ZZ |
| Resection of Stomach, Pylorus, Natural or Artificial Opening Endoscopic | 0DT78ZZ |
| Excision of Stomach, Pylorus, Open | 0DB70ZZ |
| Excision of Stomach, Pylorus, Percutaneous | 0DB73ZZ |
| Excision of Stomach, Pylorus, Percutaneous Endoscopic | 0DB74ZZ |
| Excision of Stomach, Pylorus, Natural or Artificial Opening | 0DB77ZZ |
| Excision of Stomach, Pylorus, Natural or Artificial Opening Endoscopic | 0DB78ZZ |
|  |  |
| *Hepatic Cancer Resection* |  |
| Excision of Liver, Open | 0FB00ZZ |
| Excision of Liver, Percutaneous | 0FB03ZZ |
| Excision of Liver, Percutaneous Endoscopic | 0FB04ZZ |
| Excision of Left Lobe of Liver, Open | 0FB20ZZ |
| Excision of Left Lobe of Liver, Percutaneous | 0FB23ZZ |
| Excision of Left Lobe of Liver, Percutaneous Endoscopic | 0FB24ZZ |
| Excision of Right Lobe of Liver, Open | 0FB10ZZ |
| Excision of Right Lobe of Liver, Percutaneous | 0FB13ZZ |
| Excision of Left Lobe of Liver, Percutaneous Endoscopic | 0FB14ZZ |
| Resection of Right Lobe Liver, Open Approach | 0FT10ZZ |
| Resection of Right Lobe Liver, Percutaneous Endoscopic | 0FT14ZZ |
| Resection of Left Lobe Liver, Open | 0FT20ZZ |
| Resection of Left Lobe Liver, Percutaneous Endoscopic | 0FT24ZZ |
|  |  |
| *Pancreatic Cancer Resection* |  |
| Excision of Pancreas, Open | 0FBG0ZZ |
| Excision of Pancreas, Percutaneous | 0FBG3ZZ |
| Excision of Pancreas, Percutaneous Endoscopic | 0FBG4ZZ |
| Resection of Pancreas, Open | 0FTG0ZZ |
| Resection of Pancreas, Percutaneous Endoscopic | 0FTG4ZZ |
|  |  |
| *Biliary Ductal Cancer Resection* |  |
| Excision of Common Bile Duct, Open | 0FB90ZZ |
| Excision of Common Bile Duct, Percutaneous | 0FB93ZZ |
| Excision of Common Bile Duct, Natural or Artificial Opening | 0FB97ZZ |
| Excision of Common Bile Duct, Natural or Artificial Opening Endoscopic | 0FB98ZZ |
| Resection of Common Bile Duct, Open | 0FT90ZZ |
| Resection of Common Bile Duct, Percutaneous Endoscopic | 0FT94ZZ |
| Resection of Common Bile Duct, Natural or Artificial Opening | 0FT97ZZ |
| Resection of Common Bile Duct, Natural or Artificial Opening Endoscopic | 0FT98ZZ |
| Destruction of Common Bile Duct, Natural or Artificial Opening Endoscopic | 0F598ZZ |
| Excision of Cystic Duct, Open | 0FB80ZZ |
| Excision of Cystic Duct, Percutaneous | 0FB83ZZ |
| Excision of Cystic Duct, Natural or Artificial Opening | 0FB87ZZ |
| Excision of Cystic Duct, Natural or Artificial Opening Endoscopic | 0FB88ZZ |
| Destruction of Cystic Duct, Open | 0F580ZZ |
| Destruction of Cystic Duct, Percutaneous | 0F583ZZ |
| Destruction of Cystic Duct, Natural or Artificial Opening | 0F587ZZ |
| Destruction of Cystic Duct, Natural or Artificial Opening Endoscopic | 0F588ZZ |
| Resection of Cystic Duct, Open | 0FT80ZZ |
| Resection of Cystic Duct, Percutaneous Endoscopic | 0FT84ZZ |
| Resection of Cystic Duct, Natural or Artificial Opening | 0FT87ZZ |
| Resection of Cystic Duct, Natural or Artificial Opening Endoscopic | 0FT88ZZ |
| Excision of Ampulla of Vater, Open | 0FBC0ZZ |
| Excision of Ampulla of Vater, Percutaneous | 0FBC3ZZ |
| Excision of Ampulla of Vater, Natural or Artificial Opening | 0FBC7ZZ |
| Excision of Ampulla of Vater, Natural or Artificial Opening Endoscopic | 0FBC8ZZ |
| Resection of Ampulla of Vater, Open | 0FTC0ZZ |
| Resection of Ampulla of Vater, Percutaneous Endoscopic | 0FTC4ZZ |
| Resection of Ampulla of Vater, Natural or Artificial Opening | 0FTC7ZZ |
| Resection of Ampulla of Vater, Natural or Artificial Opening Endoscopic | 0FTC8ZZ |
| Resection of Ampulla of Vater, Natural or Artificial Opening Endoscopic | 0F5C8ZZ |
| Destruction of Right Hepatic Duct, Open | 0F550ZZ |
| Destruction of Right Hepatic Duct, Percutaneous | 0F553ZZ |
| Destruction of Right Hepatic Duct, Natural or Artificial Opening | 0F557ZZ |
| Destruction of Right Hepatic Duct, Natural or Artificial Opening Endoscopic | 0F558ZZ |
| Destruction of Left Hepatic Duct, Open | 0F560ZZ |
| Destruction of Left Hepatic Duct, Percutaneous | 0F563ZZ |
| Destruction of Left Hepatic Duct, Natural or Artificial Opening | 0F567ZZ |
| Destruction of Left Hepatic Duct, Natural or Artificial Opening Endoscopic | 0F568ZZ |
| Excision of Right Hepatic Duct, Open | 0FB50ZZ |
| Excision of Right Hepatic Duct, Percutaneous | 0FB53ZZ |
| Excision of Right Hepatic Duct, Natural or Artificial Opening | 0FB57ZZ |
| Excision of Right Hepatic Duct, Natural or Artificial Opening Endoscopic | 0FB58ZZ |
| Excision of Left Hepatic Duct, Open | 0FB60ZZ |
| Excision of Left Hepatic Duct, Percutaneous | 0FB63ZZ |
| Excision of Left Hepatic Duct, Natural or Artificial Opening | 0FB67ZZ |
| Excision of Left Hepatic Duct, Natural or Artificial Opening Endoscopic | 0FB58ZZ |
| Resection of Right Hepatic Duct, Open | 0FT50ZZ |
| Resection of Right Hepatic Duct, Percutaneous | 0FT54ZZ |
| Resection of Right Hepatic Duct, Natural or Artificial Opening | 0FT57ZZ |
| Resection of Right Hepatic Duct, Natural or Artificial Opening Endoscopic | 0FT58ZZ |
| Resection of Left Hepatic Duct, Open | 0FT60ZZ |
| Resection of Left Hepatic Duct, Percutaneous | 0FT64ZZ |
| Resection of Left Hepatic Duct, Natural or Artificial Opening | 0FT67ZZ |
| Resection of Left Hepatic Duct, Natural or Artificial Opening Endoscopic | 0FT68ZZ |
|  |  |
|  |  |
|  |  |
|  |  |
|  |  |
