# Supplementary material for: Patterns and Consequences of Care Fragmentation in Post-Surgical Management of Upper Gastrointestinal and Hepatopancreatobiliary Cancers
Source: Ann Surg Oncol. 2025 Aug 20;33(1):568–77. doi: 10.1245/s10434-025-18052-8 (PMC12689737; doi:10.1245/s10434-025-18052-8)
Supplement: Supplementary file 3 — (DOCX 17 KB) [file 10434_2025_18052_MOESM3_ESM.docx]

**Supplemental Table 2: Unadjusted Outcomes of Readmission**

Outcomes reported as percentages or medians with IQR. A P-value <0.05 was considered statistically significant.

CF, Care Fragmentation; No-CF, No Care Fragmentation; IQR, Interquartile Range; USD, United States Dollar

|  |  | **UGI** |  |  | **HPB** |  |
| --- | --- | --- | --- | --- | --- | --- |
|  | ***No-CF***  (n = 7,108) | ***CF***  (n = 1,276) | ***P-value*** | ***No-CF***  (n =14,069) | ***CF***  (n =2,165) | ***P-value*** |
| **Clinical Outcomes** |  |  |  |  |  |  |
| In-Hospital Mortality (%) | 4.5 | 5.2 | 0.46 | 2.5 | 5.6 | <0.001 |
| Additional Complications (%) |  |  |  |  |  |  |
| Blood Transfusion | 7.8 | 9.5 | 0.17 | 8.0 | 9.8 | 0.05 |
| Cardiac | 3.2 | 4.4 | 0.16 | 2.5 | 5.5 | <0.001 |
| Infectious | 24.5 | 22.1 | 0.19 | 28.6 | 28.2 | 0.8 |
| Renal | 13.7 | 16.5 | 0.08 | 14.9 | 21.2 | <0.001 |
| Respiratory | 16.8 | 27.4 | <0.001 | 6.2 | 15.8 | <0.001 |
| Thromboembolic | 4.3 | 4.7 | 0.63 | 4.3 | 5.0 | 0.3 |
|  |  |  |  |  |  |  |
| **Resource Utilization** |  |  |  |  |  |  |
| Cost (USD $1000s, Median [IQR]) | 14.4 [7.8-30.6] | 11.5 [6.9-22.2] | 0.01 | 12.8 [7.2-24.7] | 11.6 [6.9-21.5] | <0.001 |
| Length of Stay (Days, Median [IQR]) | 6 [3-11] | 4 [2-7] | <0.001 | 5 [3-9] | 4 [2-7] | <0.001 |
| 30-Day Readmission (%) | 41.0 | 44.3 | 0.14 | 41.3 | 41.6 | 0.9 |
| Non-Home Discharge (%) | 20.9 | 29.0 | 0.001 | 16.4 | 27.8 | <0.001 |
