# Supplementary material for: Patterns and Consequences of Care Fragmentation in Post-Surgical Management of Upper Gastrointestinal and Hepatopancreatobiliary Cancers
Source: Ann Surg Oncol. 2025 Aug 20;33(1):568–77. doi: 10.1245/s10434-025-18052-8 (PMC12689737; doi:10.1245/s10434-025-18052-8)
Supplement: Supplementary file 4 — (DOCX 21 KB) [file 10434_2025_18052_MOESM4_ESM.docx]

**Supplemental Table 3**: **Evaluating Differential Impact of Care Fragmentation on In-Hospital Mortality Across Procedure Type**

Multivariable logistic regression evaluating the association between care fragmentation and in-hospital mortality among patients undergoing UGI or HPB cancer surgery. Interaction terms were included to assess whether this association varied by procedure type. No statistically significant interactions were observed. Logistic model outputs are reported as Adjusted Odds Ratios (AOR) with 95% confidence intervals (95% CI).

* indicates model non-convergence or no events in group.

AOR, Adjusted Odds Ratio; CI, Confidence Interval; USD, United States dollar; CF, care fragmentation; No-CF, no care fragmentation; EGJ, esophagogastric junction

|  |  | **UGI** |  |
| --- | --- | --- | --- |
|  | ***AOR*** | ***95% CI*** | ***P-value*** |
| Care Fragmentation (ref: No-CF) | 1.34 | 0.53- 3.38 | 0.53 |
|  |  |  |  |
| *Procedure Type* |  |  |  |
| Esophageal Excision | Ref | - | - |
| Esophagectomy | 1.00 | 0.42, 2.39 | 1.00 |
| *+* Autologous Tissue Substitution | 2.22 | 0.26, 18.52 | 0.46 |
| + Colonic Interposition | * | * | * |
| + Jejunal Interposition | * | * | * |
| Excision of EGJ | 1.76 | 0.73, 4.21 | 0.21 |
| Esophagogastrectomy | 2.59 | 0.86, 7.83 | 0.09 |
| Partial Gastrectomy | 0.81 | 0.34, 1.92 | 0.63 |
| Total Gastrectomy | 1.13 | 0.60, 2.14 | 0.70 |
| Antrectomy | 3.59 | 0.21, 62.15 | 0.38 |
| Pylorogastrectomy | 0.31 | 0.10, 1.02 | 0.05 |
|  |  |  |  |
| *Interaction: CF x Procedure Type* |  |  |  |
| CF x Esophagectomy | 1.10 | 0.04, 1.89 | 0.19 |
| CF x Esophagectomy + Autologous Tissue Substitution | * | * | * |
| CF x Esophagectomy + Colonic Interposition | * | * | * |
| CF x Esophagectomy + Jejunal Interposition | * | * | * |
| CF x Excision of EGJ | 0.29 | 0.04, 1.89 | 0.19 |
| CF x Esophagogastrectomy | 0.45 | 0.08, 2.45 | 0.36 |
| CF x Partial Gastrectomy | 0.95 | 0.21, 4.32 | 0.20 |
| CF x Total Gastrectomy | 0.71 | 0.24, 2.17 | 0.55 |
| CF x Antrectomy | * | * | * |
| CF x Pylorogastrectomy | 3.81 | 0.50, 29.06 | 0.20 |
|  |  |  |  |
|  |  | **HPB** |  |
|  | ***AOR*** | ***95% CI*** | ***P-value*** |
| Care Fragmentation (ref: No-CF) | 1.23 | 0.55, 2.77 | 0.62 |
|  |  |  |  |
| *Procedure Type* |  |  |  |
| Hepatic Wedge Resection | Ref | - | - |
| Right Hepatectomy | 0.25 | 0.09, 0.69 | 0.007 |
| Left Hepatectomy | 1.49 | 0.29, 7.63 | 0.64 |
| Partial Pancreatectomy | 0.66 | 0.34, 1.27 | 0.22 |
| Total Pancreatectomy | 0.98 | 0.24, 4.06 | 0.98 |
| Excision/ Resection CBD | 0.77 | 0.34, 1.74 | 0.52 |
| Excision/ Resection Cystic Duct | * | * | * |
| Excision/ Resection Ampulla | 0.71 | 0.07, 6.88 | 0.76 |
| Excision/ Resection Right Hepatic Duct | 0.28 | 0.03, 2.40 | 0.24 |
| Excision/ Resection Left Hepatic Duct | 0.47 | 0.05, 4.49 | 0.51 |
|  |  |  |  |
| *Interaction: CF x Procedure Type* |  |  |  |
| CF x Right Hepatectomy | 6.25 | 1.47, 26.59 | 0.01 |
| CF x Left Hepatectomy | 1.88 | 0.22, 16.24 | 0.56 |
| CF x Partial Pancreatectomy | 0.98 | 0.38, 2.52 | 0.97 |
| CF x Total Pancreatectomy | 1.21 | 0.17, 8.54 | 0.85 |
| CF x Excision/ Resection CBD | 0.90 | 0.26, 3.15 | 0.87 |
| CF x Excision/ Resection Cystic Duct | * | * | * |
| CF x Excision/ Resection Ampulla | * | * | * |
| CF x Excision/ Resection Right Hepatic Duct | * | * | * |
| CF x Excision/ Resection Left Hepatic Duct | * | * | * |
