# Supplementary material for: Patterns and Consequences of Care Fragmentation in Post-Surgical Management of Upper Gastrointestinal and Hepatopancreatobiliary Cancers
Source: Ann Surg Oncol. 2025 Aug 20;33(1):568–77. doi: 10.1245/s10434-025-18052-8 (PMC12689737; doi:10.1245/s10434-025-18052-8)
Supplement: Supplementary file 5 — (DOCX 17 KB) [file 10434_2025_18052_MOESM5_ESM.docx]

**Supplemental Table 4**: **Mortality by Volume Transition Patterns in Fragmented Surgical Oncologic Care**

Multivariable logistic regression examining the association between care fragmentation and in-hospital mortality, stratified by volume transitions between index and readmission hospitals. No statistically significant interactions were observed. Logistic model outputs are reported as Adjusted Odds Ratios (AOR) with 95% confidence intervals (95% CI).

AOR, Adjusted Odds Ratio; CI, Confidence Interval; USD, United States dollar; CF, care fragmentation; No-CF, no care fragmentation; EGJ, esophagogastric junction

|  |  | **UGI** |  |  | **HPB** |  |
| --- | --- | --- | --- | --- | --- | --- |
|  | ***AOR*** | ***95% CI*** | ***P-value*** | ***AOR*** | ***95% CI*** | ***P-value*** |
| *Volume Transition Pattern* |  |  |  |  |  |  |
| Low to Low | Ref | - | - | Ref | - | - |
| Low to Medium | 1.19 | 0.60, 2.36 | 0.62 | 1.84 | 0.87, 3.87 | 0.11 |
| Low to High | 1.33 | 0.51, 3.43 | 0.56 | 1.79 | 0.70, 4.54 | 0.22 |
|  |  |  |  |  |  |  |
| Medium to Low | 1.02 | 0.58, 1.77 | 0.95 | 1.88 | 0.94, 3.73 | 0.07 |
| Medium to Medium | 0.97 | 0.56, 1.65 | 0.90 | 1.45 | 0.75, 2.79 | 0.27 |
| Medium to High | 0.78 | 0.44, 1.38 | 0.39 | 1.41 | 0.74, 2.69 | 0.29 |
|  |  |  |  |  |  |  |
| High to Low | 0.87 | 0.52, 1.46 | 0.59 | 1.32 | 0.68, 2.57 | 0.41 |
| High to Medium | 0.95 | 0.58, 1.56 | 0.84 | 1.59 | 0.84, 3.00 | 0.15 |
| High to High | 0.63 | 0.39, 1.01 | 0.06 | 1.12 | 0.61, 2.05 | 0.72 |
